# Sudden Cardiac Arrest After Endurance Cycling Due to Silent Three-Vessel Coronary Artery Disease: A Case Report

**DOI:** 10.7759/cureus.102566

**Published:** 2026-01-29

**Authors:** Resha R Ganthan, Rayna Isber, Joud Fahed, Asher Gorantla, Nidal Isber

**Affiliations:** 1 Internal Medicine, Richmond University Medical Center, New York, USA; 2 Biology, Barnard College, Columbia University, New York, USA; 3 Internal Medicine, Ascension St. Agnes Medical Center, Baltimore, USA; 4 Internal Medicine, SUNY (State University of New York) Downstate Health Sciences University, New York, USA; 5 Electrophysiology, Richmond University Medical Center, New York, USA

**Keywords:** endurance exercise, multivessel coronary artery disease (mvcad), recreational athlete cardiac risk, sudden cardiac, ventricular fibrillation (vf) storm

## Abstract

Sudden cardiac arrest (SCA) during endurance exercise in middle-aged athletes is uncommon but is most frequently caused by occult coronary artery disease (CAD).

A 51-year-old, previously healthy, recreational cyclist collapsed immediately after completing a high-intensity 40-mile cycling event. He was found to be in ventricular fibrillation (VF) and achieved return of spontaneous circulation (ROSC) following prompt bystander cardiopulmonary resuscitation (CPR) and defibrillation. Initial electrocardiography and transthoracic echocardiography demonstrated no abnormalities and preserved left ventricular systolic function. Coronary angiography revealed severe three-vessel CAD, including critical proximal left anterior descending artery stenosis and chronic total occlusion of an obtuse marginal branch. The patient underwent successful coronary artery bypass grafting (CABG). Given the preserved ventricular function and a reversible ischemic cause, implantable cardioverter-defibrillator (ICD) implantation was not indicated.

This case highlights the ability of high-intensity endurance exercise to unmask advanced, asymptomatic CAD and precipitate malignant ventricular arrhythmias in middle-aged athletes, emphasizing the importance of individualized cardiovascular risk assessment and emergency preparedness at endurance events.

## Introduction

Endurance sports, such as long-distance cycling, are widely regarded as cardioprotective and are associated with substantial reductions in cardiovascular morbidity and mortality [1-3]. Regular aerobic exercise improves cardiorespiratory fitness and favorably modifies traditional cardiovascular risk factors, leading to widespread promotion of endurance activities, particularly among middle-aged and older adults [2-4]. Paradoxically, vigorous endurance exercise in this population may precipitate acute cardiovascular events by unmasking previously silent coronary artery disease [5,6]. Sudden cardiac arrest during or immediately after endurance events, although rare, remains a devastating presentation of occult atherosclerotic disease [5-7]. In athletes over 35 years of age, the predominant mechanism of exercise-related sudden cardiac arrest is atherosclerotic coronary artery disease rather than inherited structural or electrical disorders [8]. We describe the case of a previously healthy 51-year-old recreational cyclist who experienced out-of-hospital sudden cardiac arrest immediately after completing a large-scale endurance cycling event and was subsequently found to have severe three-vessel coronary artery disease.

## Case presentation

A 51-year-old male with no significant past medical history completed the 40-mile Five Boro Bike Tour in approximately 2 hours, substantially faster than the average finish time of 4 to 5 hours, indicating a high-intensity exertion level. Upon reaching the finish line, he collapsed suddenly. He had no memory of the event and reported no preceding symptoms such as chest pain, dizziness, or palpitations.

Baseline cardiovascular risk factors were unremarkable, with no known history of hypertension, hyperlipidemia, diabetes, or smoking, and no prior cardiovascular testing.

Bystander cardiopulmonary resuscitation was initiated immediately. Paramedics arrived to find him in ventricular fibrillation and performed advanced cardiac life support. He was intubated on scene and received two external defibrillator shocks, achieving return of spontaneous circulation. The estimated collapse-to-first-defibrillation time was one minute, and return of spontaneous circulation (ROSC) occurred approximately three minutes after collapse. He was transported to the hospital, admitted to the intensive care unit for post-cardiac arrest care, and later extubated with full neurologic recovery.

**Figure 1:**
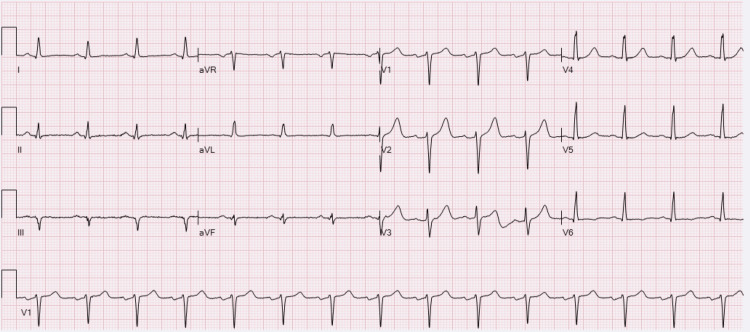
Admission electrocardiogram demonstrating normal sinus rhythm without ST-segment changes suggestive of acute ischemia

Initial electrocardiography demonstrated a normal sinus rhythm with normal intervals and no ischemic ST-segment changes (Figure 1). Transthoracic echocardiography showed a preserved left ventricular ejection fraction of 60-65% and no regional wall-motion abnormalities. High-sensitivity troponin peaked at 416 ng/L post-return of spontaneous circulation. The magnitude and timing of elevation were felt to reflect acute ischemic myocardial injury, with possible contribution from resuscitation-related myocardial stress.

**Figure 2:**
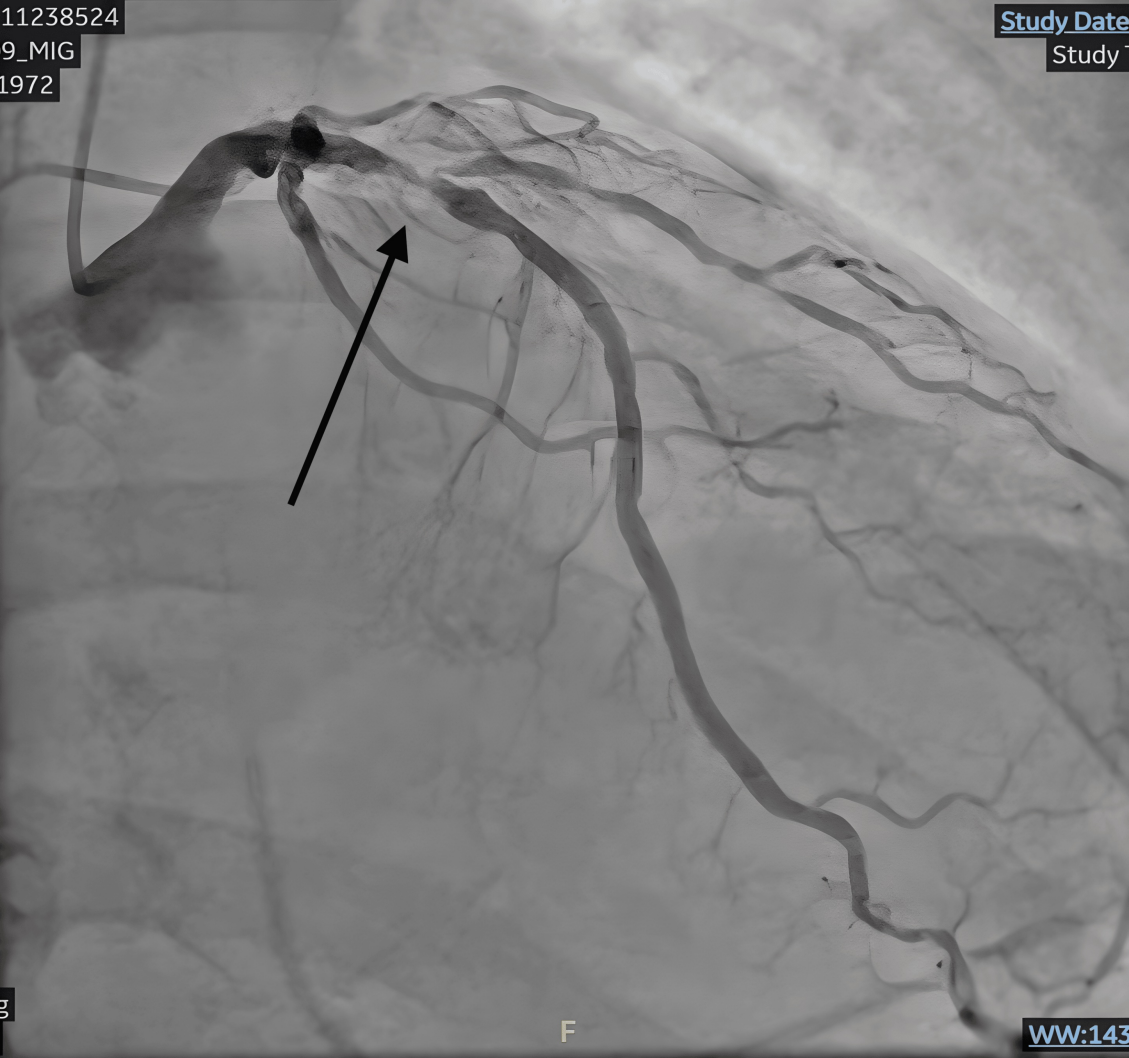
Critical proximal left anterior descending artery stenosis Coronary angiogram demonstrating a heavily calcified, 99% proximal, left anterior descending artery stenosis.

**Figure 3:**
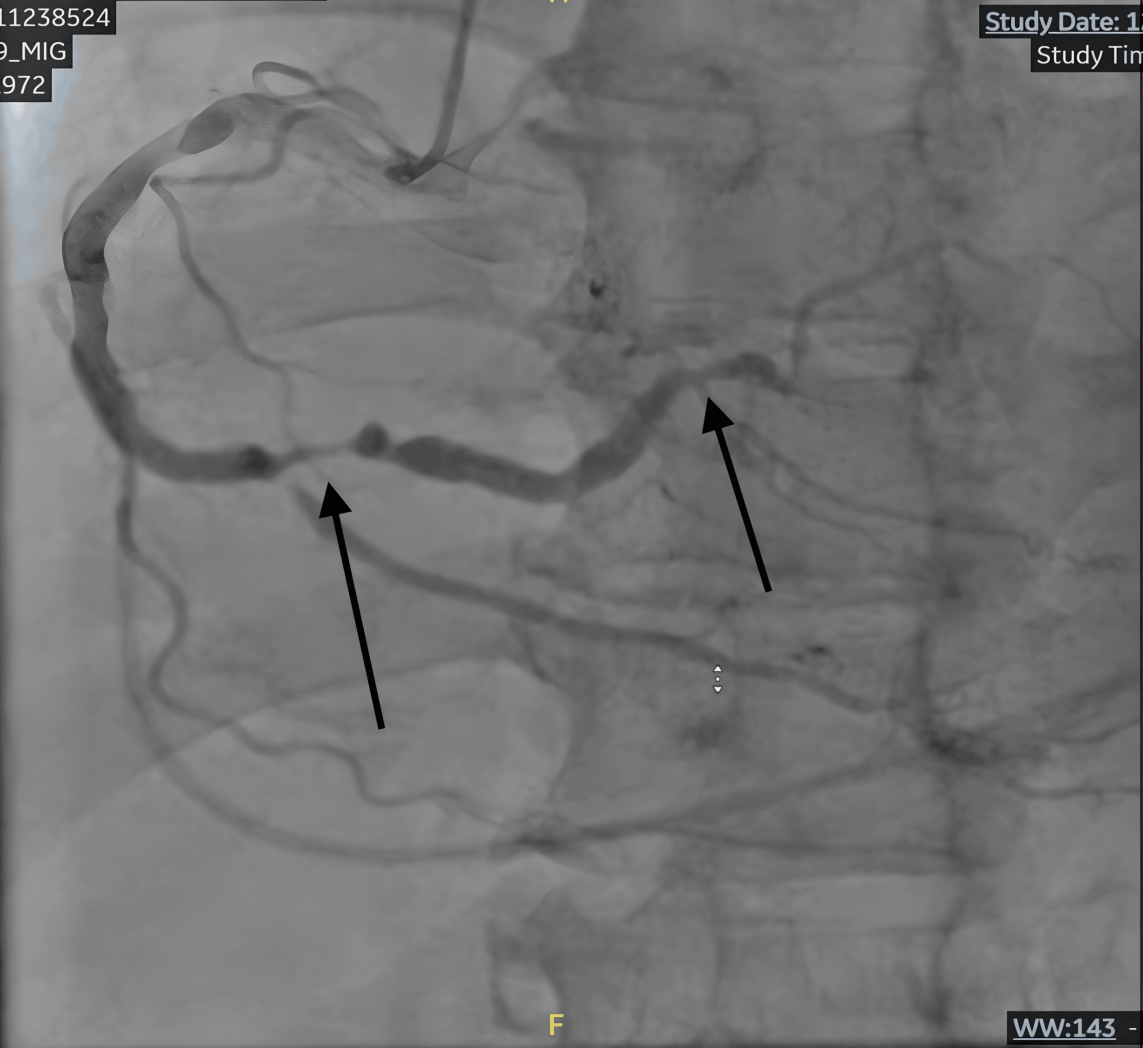
Chronic total occlusion of the first obtuse marginal branch and severe stenoses of the second obtuse marginal and distal right coronary artery (arrows) on coronary angiography; RAO projection RAO: right anterior oblique

Coronary angiography demonstrated severe obstructive three-vessel coronary artery disease, including a critical, 99% calcified, proximal left anterior descending artery stenosis (Figure 2), chronic total occlusion of the first obtuse marginal branch, severe stenosis of the second obtuse marginal branch, and diffuse right coronary artery disease with high-grade (90-95%) distal bifurcation stenoses (Figure 3). The left main coronary artery had only minimal non-obstructive disease. Coronary angiography was performed approximately 1.5 hours after return of spontaneous circulation.

The patient underwent successful coronary artery bypass grafting. His postoperative course was uncomplicated, with no recurrent ventricular arrhythmias, stroke, renal dysfunction, or heart failure. He was discharged on high-intensity statin therapy, aspirin, clopidogrel, and guideline-directed medical therapy, and was enrolled in structured cardiac rehabilitation with a graduated return-to-exercise plan. Given preserved left ventricular systolic function and a reversible ischemic cause of ventricular fibrillation, implantable cardioverter-defibrillator implantation was not indicated.

## Discussion

This case illustrates how vigorous physical exertion during endurance events can unmask previously silent but advanced coronary artery disease. Despite being asymptomatic and having a normal baseline electrocardiogram and preserved left ventricular systolic function, this patient experienced sudden cardiac arrest immediately after high-intensity exertion and was found to have severe three-vessel coronary artery disease. This presentation highlights a recognized but often underappreciated phenomenon in endurance athletes, in whom high functional capacity may mask extensive atherosclerotic disease until a catastrophic event occurs.

Coronary artery disease remains the leading cause of exercise-related sudden cardiac arrest and death in individuals older than 35 years, accounting for approximately 75-80% of cases [1]. Multiple reports describe endurance athletes presenting with exertional ventricular arrhythmias who are subsequently diagnosed with advanced multivessel coronary artery disease despite no prior symptoms [2-4]. Silent myocardial ischemia in physically conditioned individuals has been well-described and may delay recognition of clinically significant disease [5]. Endurance training-related coronary calcification and vascular remodeling have been proposed as contributors to plaque burden and symptom attenuation, potentially delaying clinical detection in highly trained individuals [9,10].

Population-level data further contextualize this case. The Race Associated Cardiac Event Registry analysis of more than 29 million US marathon and half-marathon participants demonstrated that while the incidence of cardiac arrest remained stable over time, survival improved substantially, approaching two-thirds of cases [7]. Coronary artery disease emerged as the most common identifiable etiology, particularly among older male athletes, aligning closely with this patient’s presentation.

Improved survival was strongly associated with rapid bystander cardiopulmonary resuscitation, shockable initial rhythms, and prompt defibrillation [7]. In this case, the short collapse-to-defibrillation interval likely contributed substantially to the patient’s complete neurologic recovery.

From a pathophysiologic perspective, intense exertion leads to a marked increase in myocardial oxygen demand, shear stress, and catecholamine release, which may precipitate ischemia-triggered ventricular arrhythmias through either acute plaque rupture with thrombosis or demand ischemia in the setting of fixed obstructive disease [6]. The presence of severe, calcified multivessel coronary artery disease without an alternative arrhythmogenic substrate supports demand ischemia as the most likely trigger in this case [7,11]. Completing the cycling event at approximately twice the average pace likely exceeded this patient’s ischemic threshold, precipitating ventricular fibrillation.

Current professional society guidelines emphasize individualized cardiovascular risk assessment rather than routine screening of asymptomatic athletes [1,2]. Targeted evaluation in selected middle-aged endurance athletes, such as those with traditional cardiovascular risk factors, high cumulative exercise exposure, or participation in high-intensity competitive events, may reasonably include coronary artery calcium scoring, functional stress testing, or cardiology referral as part of shared decision-making [12].

Despite experiencing ventricular fibrillation, this patient maintained normal left ventricular systolic function and had a clearly reversible ischemic etiology. Consistent with contemporary guideline recommendations, ICD implantation for secondary prevention was not indicated in the absence of residual structural heart disease or ongoing arrhythmic risk following revascularization [13]. He underwent successful coronary artery bypass grafting without complication.

As a single-case observation, this report cannot define screening thresholds or establish causality; however, it provides important educational value by illustrating a clinically relevant mechanism of exertion-related sudden cardiac arrest and informing risk discussion in similar patient populations.

## Conclusions

We report the case of a previously healthy, middle-aged recreational cyclist who suffered sudden cardiac arrest immediately after a high-intensity endurance event and was found to have severe triple-vessel coronary artery disease. This case underscores the potential for silent advanced coronary artery disease in middle-aged athletes, the limitations of routine screening, and the importance of individualized cardiovascular risk assessment and shared decision-making. It further highlights key considerations for post-revascularization prognosis, secondary prevention, and safe return-to-exercise counseling. The favorable neurologic outcome reinforces the life-saving role of rapid bystander cardiopulmonary resuscitation and early defibrillation, emphasizing the continued need for robust emergency preparedness at endurance sporting events.
